# Enforcement agencies and smoke-free policy compliance: An observational study in Qingdao, China

**DOI:** 10.18332/tid/133635

**Published:** 2021-04-12

**Authors:** Connie Hoe, Hanaa Ahsan, Xuejuan Ning, Xiaojing Wang, Dafei Li, Kathy Wright, Ryan D. Kennedy

**Affiliations:** 1 Institute for Global Tobacco Control, Johns Hopkins Bloomberg School of Public Health, Baltimore, United States; 2 Vital Strategies, Jinan, China; 3 International Union Against Tuberculosis and Lung Disease, New York City, United States

**Keywords:** China, compliance, enforcement, smoke-free policy, tobacco control

## Abstract

**INTRODUCTION:**

In recent years, tobacco control policy initiatives have emerged at the subnational level in China. In 2013, for example, Qingdao enacted a 100% smoke-free policy that gave regulatory authority to multiple enforcement agencies. Given that little is known about the extent of smoke-free policy compliance in smaller Chinese cities, this study assessed compliance with Qingdao’s Tobacco Control Regulation and whether compliance differed by enforcement agency.

**METHODS:**

A cross-sectional observational study was undertaken between October and November 2018. Venues were selected based on enforcement agency and included restaurants, retail stores, schools, government buildings, hospitals, business offices, and other hospitality venues. Comprehensive lists of venues were identified where they existed, and a random sample of venues were subsequently selected. For venue categories for which there were no comprehensive lists, a walking protocol was used. Observational data included evidence of smoking, the presence of no-smoking signage, and designated smoking areas (DSAs). Descriptive statistics were obtained. Subsequently, logistic regression models were used to determine the association between enforcement agency and policy compliance.

**RESULTS:**

A total of 694 venues were observed. For all venue types, 64.7% were compliant with the composite indicator ‘evidence of smoking’. Findings also showed that smoke-free compliance varied by enforcement agency (p<0.001). Venues with evidence of smoking and not posting of no-smoking signs at the main entrance were lowest among venues that fall under Public Security Bureau. Compliance with posting no-smoking signs inside was lowest in venues that fall under the Industry and Commercial Administration (I&C). While Qingdao’s smoke-free policy prohibits DSAs, our findings showed that 2% of venues that fall under the jurisdiction of I&C had DSAs.

**CONCLUSIONS:**

An effective coordination mechanism that can ensure a consistent and standardized approach is urgently needed in Qingdao. With such a concerted effort, it will be possible to achieve the target of 100% smoke-free indoor places in Qingdao.

## INTRODUCTION

Secondhand smoke (SHS) exposure is a pressing public health concern as it serves as an important risk factor for an array of non-communicable diseases, including ischemic heart disease, lung cancer, and stroke^1^. There is no level of SHS exposure that is considered safe; only 100% smoke-free environments can effectively prevent SHS exposure^1^. Accordingly, the World Health Organization (WHO) recommends the passage and implementation of comprehensive national smoke-free legislations in order to fully protect people from SHS^2^.

China leads the world in both tobacco production, manufacturing and consumption; one-third of the world’s cigarette smokers live in China^3^. While the country ratified the Framework Convention for Tobacco Control in 2005^4^, it currently does not have a comprehensive national smoke-free policy, despite the fact that the FCTC requires states that are parties to the convention to implement comprehensive smoke-free laws within 5 years of ratification^5^. Recent findings from the 2018 Global Adult Tobacco Survey (GATS) showed that 26.6% of the population are current smokers (50.5% among males and 2.1% among females)^6^. It is also estimated that 70% of Chinese adults are regularly exposed to SHS. Further, tobacco products result in over 1.3 million tobacco-related deaths in China each year, of which 100000 are due to SHS^7^.

In recent years, tobacco control policy initiatives have emerged in China at the subnational level. All tier 1 cities (cities that have higher Gross Domestic Product, better infrastructure and more political resources) namely Beijing, Shanghai and Shenzhen have implemented local smoke-free policies with promising results. Xiao et al.^8^, for example, showed that less smoking was observed in restaurants in Beijing one month after the Beijing Smoking Control Regulation took effect in 2015 (40.3% to 14.8% of restaurants). Similarly, in Shenzhen, following the enactment of the city’s smoke-free law in 2014, there was a significant reduction in the prevalence of observed smoking in hospitals, hotels, and indoor waiting areas for public transport^9^. One of the key factors that contributed to these successes was enhanced enforcement efforts guided by effective coordination among the various enforcement agencies^10^. Enforcement is a critical component of policy implementation^11^ and can be defined as means employed to gain compliance with the law^12^. In countries like China where a national comprehensive smoke-free law does not exist and in many of its cities tobacco control is governed by multiple enforcement agencies, coordination is particularly critical and can serve as a key challenge to the implementation of smoke-free policy^13^.

In 2013, Qingdao, a new first-tier city (a tier immediately below tier 1) in eastern Shandong province, enacted its Tobacco Control Regulation, which went into effect in September of the same year^14^. This policy bans smoking in all indoor public and workplaces and designates multiple agencies, including the: Food and Drug Administration (FDA), Health Commission, Public Security Bureau, Education Bureau and Industry and Commercial Administrations (I & C) to enforce this regulation. The FDA is charged with the supervision of catering service establishments; the Health Commission manages venues that do not fall under other jurisdictions such as hospitals, government buildings and business office buildings; the Public Security Bureau is responsible for establishments that provide accommodation, beauty salons, leisure and entertainment services, and internet services; the Education Bureau oversees educational and training institutions; and the I&C Administration manages shopping malls, supermarkets, and other shopping places^14^.

While compliance with smoke-free policies has been assessed in tier 1 cities, as mentioned above, few have focused on cities in other tiers like Qingdao which has less capacity and resources to enforce its smoke-free policy. One study did find that Qingdao’s smoke-free policy was associated with a reduction in hospitalization from acute myocardial infraction and stroke^15^. However, Xu et al.^16^ showed that smoking-attributable cancer remains significant in the city despite the presence of tobacco control measures.

In light of these gaps, the primary objectives of this observational study were to assess compliance with Qingdao’s Tobacco Control Regulation and to explore whether compliance differed by enforcement agencies in order to guide future interventions.

## METHODS

### Study design

A cross-sectional observational study was undertaken between October and November 2018 in Qingdao to meet the study objectives. A mix of urban (Laoshan, Shibei, Shinan) and suburban (Huangdao, Pingdu) districts in Qingdao were selected as the study area. These districts were chosen to reflect geographical diversity. Venue types were selected based on enforcement agency (e.g. if it falls under the jurisdiction of the Food and Drug Administration, Health Commission, Public Security Bureau, Education Bureau and Industry and Commercial Administrations), review of Qingdao’s policy, and input from in-country collaborators. Sample size was determined based on minimal sample size for group comparison. With a power of 0.95 and a medium effect size of 0.5, approximately 100 venues were needed per enforcement agency. The study was approved as non-human subjects research by the authors’ University Institutional Review Board.

### Training

A data collectors training was held immediately before data collection. The first day of the training introduced the data collectors to Qingdao’s smoke-free policy and provided them with an overview of the data collection protocol and the observation tools. The subsequent days comprised of field practice sessions, where data collectors had the opportunity to practice data collection in the field. A debriefing session was held at the end of the day to address questions that emerged.

### Data collection

Comprehensive lists of venues were identified where they existed for each venue. Subsequently, a random sample of venues was selected for observations. This was done by sorting the comprehensive list of venues using a random seed. We then picked the first n (number of venues needed) of each type of venue. In the case of children and maternity hospitals, a census rather than random sampling approach was used as only a total of 25 were identified in Qingdao. For venue categories such as business office buildings, pubs/bars, game rooms, billiard rooms, comprehensive hospitality venues, karaoke, clubs, and retail stores, for which there were no comprehensive lists, a walking protocol adapted from the ‘How-to’ Guide for Conducting Compliance Studies^17^ was used. Data collectors were instructed to arrive at a pre-identified hub and walk to the right for 10 minutes and observe all the relevant venues (business office buildings, pubs/bars, game rooms, billiard rooms, comprehensive hospitality venues, karaoke, clubs, and retail stores) on the way. Once the initial 10 minutes were over, they were instructed to take another right and walk for 10 minutes and observe all the relevant venues on the way. They were asked to repeat the previous step three more times until they returned to the original hub. If the number of venues that the data collectors were required to observe had not been reached, they were asked to proceed on to the next hub and follow the steps above.

Following a standardized protocol, trained data collectors worked in pairs (one male and one female) and visited the study venues during regular business hours when people were most likely to be present. For example, data collectors were instructed to visit internet bars after 18:00 (Table 1). In each of the venues, data collectors observed the main floor and two additional floors if the venue was multistoried, bathrooms, elevators, hallways, and stairways, when applicable. Data collectors corroborated their observations and entered findings into an observation form that was uploaded to a mobile data collection application. This form was developed based on the ‘How-to’ Guide for Conducting Compliance Studies^17^ and local policy. Questions pertained to the presence or absence of smoker(s), cigarette butts, ashtrays or other instruments, no-smoking signage at the main entrance and inside the venues, as well as designated smoking areas/rooms.

**Table 1:** Time of observation by venue type, Qingdao 2018 (N=694)

*Enforcement agency*	*Type of venue*	*Time of observation*
**Public security bureau**	Hotels	8:00–22:00
	Pubs/bars	After 18:00
	Game rooms	After 18:00
	Billiard rooms	After 18:00
	Comprehensive hospitality venue	After 18:00
	Karaoke	After 18:00
	Internet bars	After 18:00
	Clubs	After 18:00
**Health commission**	Government buildings	9:00–17:00
	Children and maternity hospitals	8:00–17:00
	General hospitals	8:00–17:00
	Business office buildings	09:00–17:00
**FDA**	Restaurants	11:30–13:00, 17:30–20:00
**Education bureau**	Universities	11:00–14:00
	Elementary and middle schools	11:00–14:00
**Industry and commercial administration**	Shopping malls & retail stores	09:00–17:00

### Measures

The dependent variables in this study were: 1) compliance with no ‘evidence of smoking’; 2) compliance with no-smoking sign at the main entrance; and 3) compliance with no-smoking sign inside the venue. All three variables were coded dichotomously: 1=compliant and 0=not compliant. A venue was considered compliant with the composite indicator assessing ‘evidence of smoking’ if it met the following criteria: 1) no one is observed smoking inside; 2) no cigarette butts; and 3) no ashtrays or other instruments used to hold cigarette ash inside. A venue was considered non-compliant if any one of the three criteria was not met.

The independent variable in this study was enforcement agency and categorized as: 1) Public Security Bureau, 2) Health Commission, 3) FDA, 4) Education Bureau, and 5) Industry and Commercial Administration.

### Statistical analysis

Descriptive statistics were first obtained to show compliance by venue type and enforcement agency. Subsequently, logistic regression models were used to determine the association between enforcement agency and smoke-free policy compliance. All data were analyzed using Stata version 14.1 statistical software (StatCorp., College Station, TX, USA).

## RESULTS

A total of 694 venues were observed in Qingdao, of which 22.1% (n=153) fell under the Public Security Bureau; this included hotels (9.9%), pubs/bars (1.6%), game rooms (0.4%), billiard rooms (1.0%), comprehensive hospitality venue (1.3%), karaoke (3.5%), internet bars (3.0%), and clubs (1.3%). Another 29.3% (n=203) of the total venues fell under the Health Commission, and included government buildings (8.8%), children and maternity hospitals (2.0%), general hospitals (9.8%), and business office buildings (8.7%). About 22% (n=152) fell under FDA; these venues were all restaurants (22%). About 18% (n=123) fell under Education Bureau and included Universities (8.8%) and Elementary and Middle Schools (8.9%). Finally, approximately 9% (n=63) fell under Industry and Commercial Administration and were all shopping malls and retail stores (9.1%) (Table 2).

**Table 2:** Sample size by enforcement agency and venue type, Qingdao 2018 (N=694)

*Enforcement agency*	*Type of venue*	*Sample size n (%)*
**Public security bureau**	Hotels	69 (9.94)
	Pubs/bars	11 (1.59)
	Game rooms	3 (0.43)
	Billiard rooms	7 (1.01)
	Comprehensive hospitality venue	9 (1.30)
	Karaoke	24 (3.46)
	Internet bars	21 (3.03)
	Clubs	9 (1.30)
	Subtotal	153 (22.05)
**Health commission**	Government buildings	61 (8.79)
	Children and maternity hospitals	14 (2.02)
	General hospitals	68 (9.80)
	Business office buildings	60 (8.65)
	Subtotal	203 (29.25)
**FDA**	Restaurants	152 (21.90)
	Subtotal	152 (21.90)
**Education bureau**	Universities	61 (8.79)
	Elementary and middle schools	62 (8.93)
	Subtotal	123 (17.72)
**Industry and commercial administration**	Shopping malls and retail stores	63 (9.08)
	Subtotal	63 (9.08)
	Total	694

**Figure 1:**
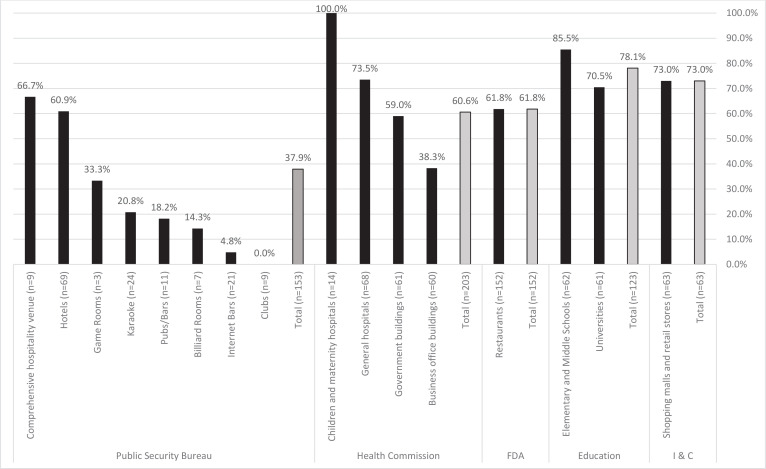
Compliance with the composite indicator ‘evidence of smoking’ by venue type and enforcement agency, Qingdao 2018 (N=694)

Findings showed that 64.7% of all venues were compliant with the composite indicator ‘evidence of smoking.’ Results, however, varied across the different venue types; all children and maternity hospitals, 85.5% of elementary and middle schools, 73.5% of general hospitals, and 73.0% of shopping malls and retail stores were compliant with the composite indicator. By comparison, 18.2% of pubs/bars, 14.3% billiard rooms, 4.8% of internet bars and none (0%) of the clubs were compliant (Figure 1). As seen in Table 3, the type of enforcement agency was associated with compliance (p<0.001). The odds of compliance with the composite indicator ‘evidence of smoking’ were significantly higher for venues that fall under the jurisdiction of Health Commission (OR=2.5; 95% CI: 1.6–3.9), FDA (OR=2.7; 95% CI: 1.7–4.2), Education Bureau (OR=5.8; 95% CI: 3.8–10.0), and Industry and Commercial Administration (OR=4.4; 95% CI: 2.3–8.4) than the odds for venues that fall under the jurisdiction of the Public Security Bureau.

**Table 3:** Association between enforcement agency and smoke-free compliance, Qingdao 2018 (N=694)

*Enforcement agency*	*Compliant with no ‘evidence of smoking’ OR (95% CI)*	*Compliant with no-smoking sign at the main entrance OR (95% CI)*	*Compliant with no-smoking sign inside the venue OR (95% CI)*
Public security bureau (Ref.)	1.0	1.0	1.0
Health commission	2.5 (1.6–3.9)***	4.7 (2.4–9.3)***	6.1 (3.5–10.5)***
FDA	2.7 (1.7–4.2)***	1.3 (0.6–3.0)	0.9 (0.6–1.5)
Education bureau	5.8 (3.4–10.0)***	2.4 (1.1–5.2)*	1.4 (0.9–2.3)
Industry and commercial administration	4.4 (2.3–8.4)***	1.9 (0.7–4.9)	0.4 (0.2–0.8)**

* p<0.05,

** p<0.01,

*** p<0.001.

Ref.: reference.

Less than 50% of all venue types had no-smoking signs posted at their main entrances and none of the pubs, bars, game rooms and comprehensive hospitality venues observed had no-smoking signs posted at their main entrances (Supplementary file). Compliance with this indicator was 17.3% for all venue types. While all children and maternity hospitals as well as comprehensive hospitality venues had no-smoking signs inside, only 33.3% of game rooms had no-smoking signs inside (Supplementary file). The type of enforcement agency was associated with compliance with the placement of no-smoking signs at the main entrance (p<0.001) and inside the venue (p<0.001). The odds of compliance with the placement of no-smoking signs at the main entrance were significantly higher for venues that fall under the jurisdiction of Health Commission (OR=4.7; 95% CI: 2.4–9.3) and Education Bureau (OR=2.4; 95% CI: 1.1–5.2) than the odds for venues that fall under the jurisdiction of the Public Security Bureau. The odds of compliance with the placement of no-smoking signs inside the venue were significantly higher for venues that fall under the jurisdiction of Health Commission (OR=6.1; 95% CI: 3.5–10.5) than the odds for venues that fall under the jurisdiction of the Public Security Bureau. However, the odds of compliance with the placement of no-smoking signs inside the venue were significantly lower for venues that fall under the jurisdiction of Industry and Commercial Administration (OR=0.4; 95% CI: 0.2–0.8) than the odds for venues that fall under the jurisdiction of the Public Security Bureau (Table 3).

While Qingdao’s smoke-free policy prohibits designated smoking rooms and areas, our findings showed that 2% of shopping malls and retail stores had designated smoking areas where smokers are allowed to smoke. This venue type falls under the jurisdiction of the Industry and Commercial Administration.

## DISCUSSION

This study assessed compliance with Qingdao’s Tobacco Control Regulation. Findings showed that smoke-free compliance varied by venue type as well as by enforcement agency. All children and maternity hospitals and about 86% of elementary and middle schools were found to be compliant with the composite indicator ‘evidence of smoking’. While 86% is higher than the percentage of compliance at other venue types observed, this finding is still concerning as it reveals that not all of the elementary and middle schools in Qingdao are smoke-free, pointing to the urgent need to fully implement Qingdao’s Tobacco Control Regulation to protect the health of Chinese children and youth.

A much lower percentage of hospitality venues such as clubs (0%) and internet bars (5%) were found to be compliant. This finding is consistent with existing studies in China as well as other countries including India and Turkey^9,18,19^. In Harbin, China, for example, compliance was found to be lowest in restaurants, internet bars and pubs^20^. It is important to note that internet bars are a venue type of unique concern to Asian countries. In China, it continues to be a thriving industry with an estimated 135000 legal internet cafes country-wide. This number does not include the many illegal operations^21^. While these venues used to be frequented by youth without personal computers, today, with growing personal computer ownership, these internet bars have evolved into venues primarily for gaming and patronized largely by young people. Existing studies also show that internet bar visits have been associated with smoking as well as drinking^22-24^. This finding has implications for other Asian countries with a flourishing and/or emerging internet bar scene.

Findings also varied by enforcement agency. Compliance with the composite indicator ‘evidence of smoking’ as well as not posting of no-smoking signs at the main entrance were found to be lowest among venues that fall under the jurisdiction of the Public Security Bureau. This could be due to the fact that this enforcement agency is primarily charged with enforcing the smoke-free regulation in hospitality venues and that the main focus of the Public Security Bureau is to crackdown on crime. In fact, based on conversations with enforcement officers, one of the key barriers across all agencies is that the implementation of tobacco control policies is not the main work area of any given agency (personal communication, 2020). Compliance with posting no-smoking signs inside was lowest in venues that fall under the jurisdiction of the Industry and Commercial Administration. Moreover, while designated smoking areas are prohibited by Qingdao’s regulation, 2% of the venues that fall under this enforcement agency had designated smoking areas. Qualitative research is needed to help further explore the specific barriers that each enforcement agency faces while implementing the smoke-free policy.

This study points to the utility of observing compliance by each enforcement agency in countries like China that utilize multiple enforcement agencies to implement smoke-free policy. Findings highlight the opportunity for Qingdao to enhance enforcement, particularly for hospitality venues, remove designated smoking areas, and establish a more effective coordination mechanism in order to fully protect citizens from SHS in all public places and workplaces. A coordination mechanism led by a politically powerful leader (e.g. Mayor or Vice Mayor) might be particularly effective in mobilizing all enforcement agencies. These efforts could be complemented by the launching of social marketing campaigns to raise awareness on the harms of smoking and SHS among the general public and venue owners, particularly hospitality venue owners. A study conducted by Kegler et al.^25^ found that knowledge about the harms of SHS is tied to support for smoke-free policies in China. However, the most recent GATS data showed that this knowledge is currently fairly low in the country^6^. It is also important to note that the role of the general public in monitoring smoke-free implementation is crucial^26^. Individuals may engage in ‘whistleblowing’ and/or filing complaints to help strengthen the monitoring of noncompliance and assist enforcement agencies in identifying ‘hot spots’.

### Limitations

There are some limitations associated with this study. Our results might not be generalizable to all of Qingdao as we only sampled from five of seven districts. Observers only spent an average of 10 minutes inside each venue, as such, some evidence of smoking might have been missed. Moreover, observations were only carried out in government buildings affiliated with the Executive Branch and Government Affiliated Institution as we were not able to obtain access to other types of government buildings. Finally, it might be possible that venues differ in self-enforced compliance. Child and maternity hospitals, for example, may be more compliant regardless of which enforcement agency is responsible. Future studies could explore this by obtaining data related to self-compliance to control for these factors.

## CONCLUSIONS

Qingdao is one of the new first-tier cities in China to enact a comprehensive smoke-free policy, which bans smoking in all indoor public places and workplaces. While these polices are a victory for public health, this study showed that there is an opportunity to improve smoke-free compliance. Given that multiple agencies are charged with enforcing the smoke-free policy and findings from this study revealed that compliance varies across enforcement agencies, an effective coordination mechanism that can mobilize all enforcement agencies and ensure a consistent approach city-wide is needed. Moreover, the utilization of standardized protocols by all enforcement agencies as well as standardized training of enforcement officers are also critical. This study points to the utility of assessing compliance by enforcement agencies in countries like China in order to better tailor interventions to establish a uniform enforcement approach, ensure 100% smoke-free environments, and protect people from exposure to SHS.
