# Supplementary material for: Enforcement agencies and smoke-free policy compliance: An observational study in Qingdao, China
Source: Tob Induc Dis. 2021 Apr 12;19:26. doi: 10.18332/tid/133635 (PMC8040544; doi:10.18332/tid/133635)
Supplement: Supplementary file 1 [file TID-19-26-s1.pdf]

Compliance with Posting No-Smoking Sign at the Main Entrance by Venue Type and Enforcement Agency

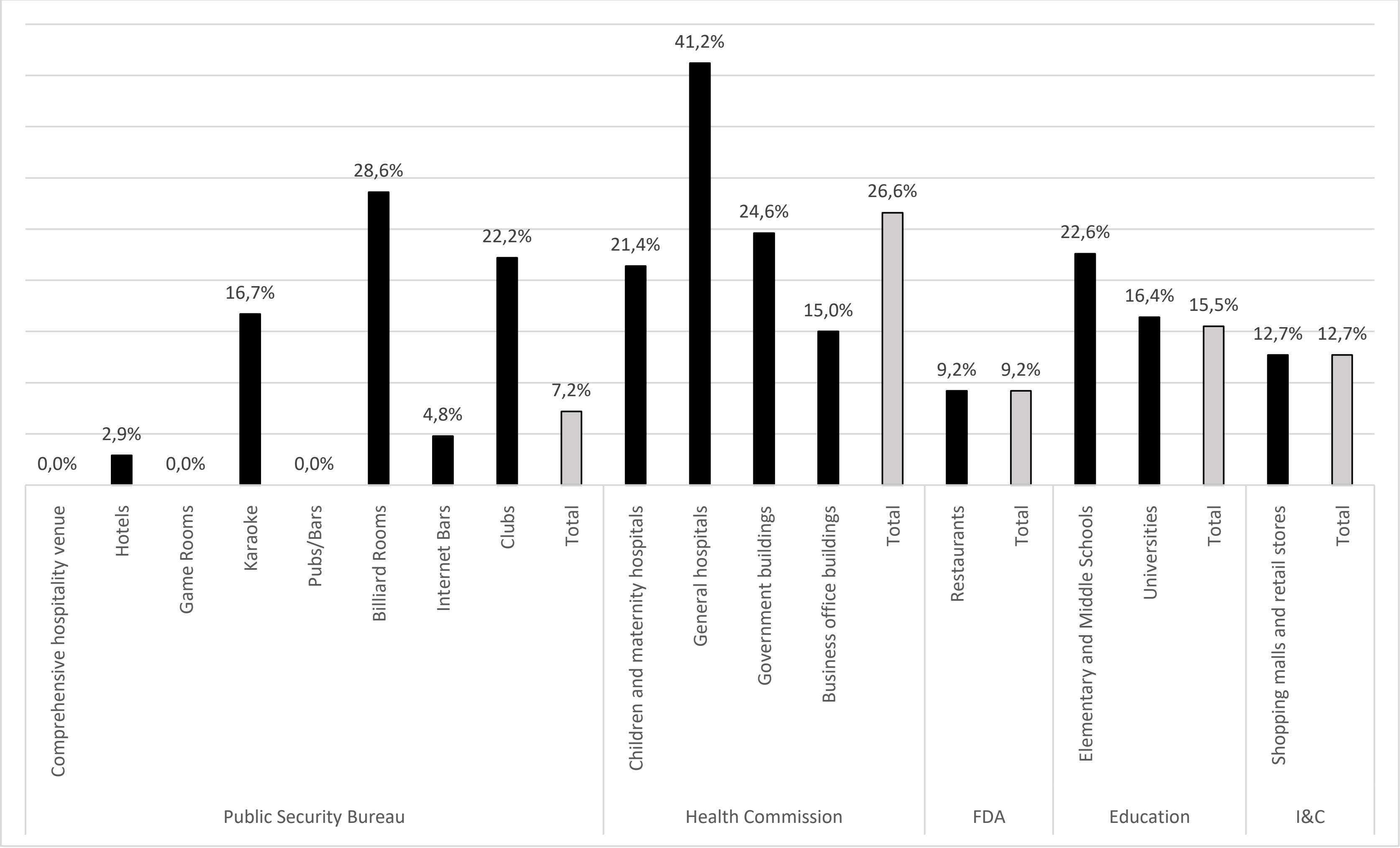

Compliance with Posting No-Smoking Sign Inside the Venue by Venue Type and Enforcement Agency

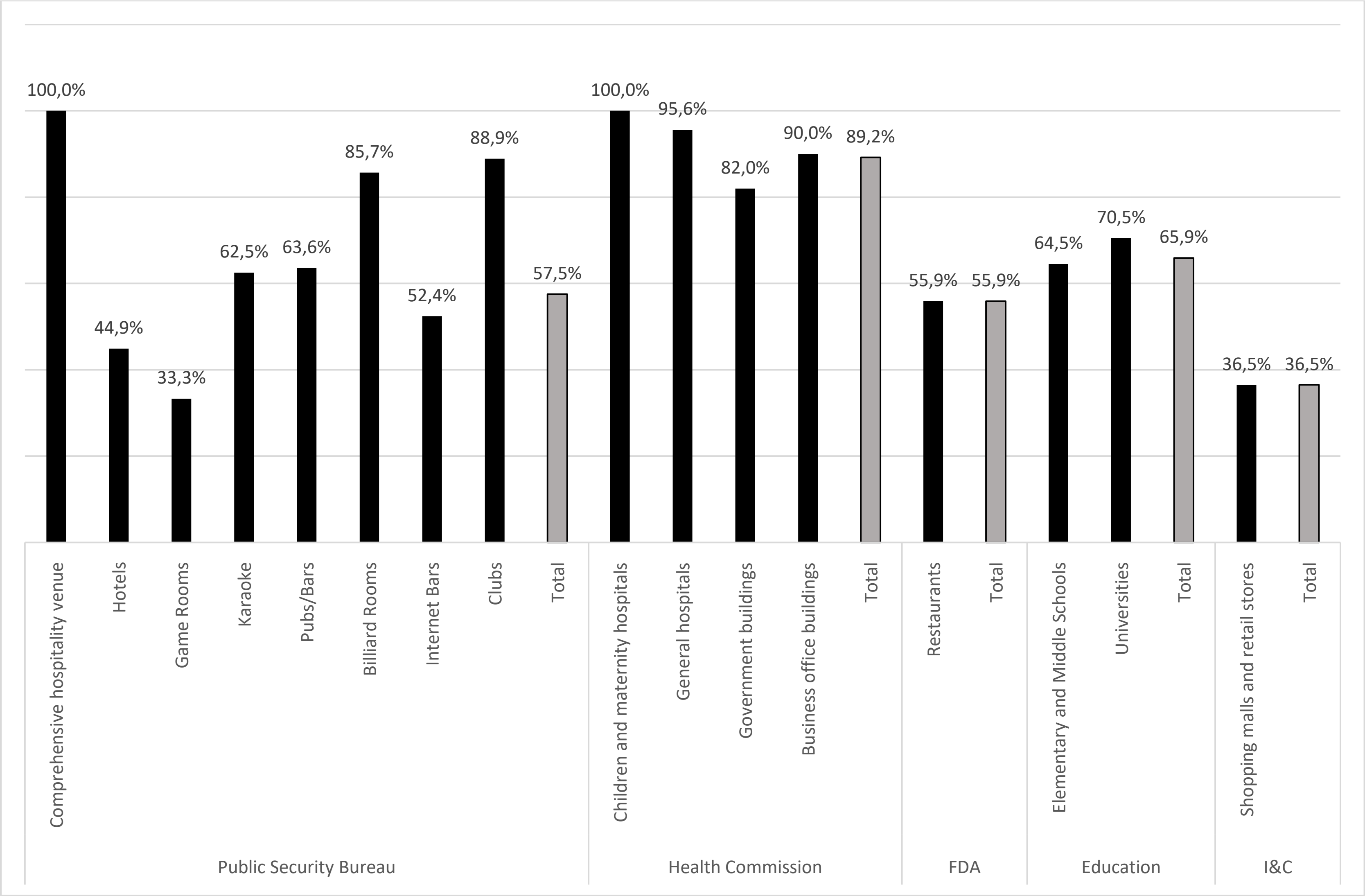

© 2021 Hoe C. et al.
